# Pharmacy sales data versus ward stock accounting for the surveillance of broad-spectrum antibiotic use in hospitals

**DOI:** 10.1186/1471-2288-11-166

**Published:** 2011-12-13

**Authors:** Jon B Haug, Randi Myhr, Åsmund Reikvam

**Affiliations:** 1 Department of Infectious Diseases, Oslo University Hospital Aker, Trondheimsveien 235, NO-0514 Oslo, Norway; 2 Regional medicines information and pharmacovigilance centre (RELIS), Oslo University Hospital Ullevål, Kirkeveien 166, NO-0407 Oslo, Norway; 3 Institute of Pharmacology, Institute of Clinical Medicine, Faculty of Medicine, University of Oslo, Sognsvannsveien 20, NO-0424 Oslo, Norway

## Abstract

**Background:**

Antibiotic consumption in hospitals is commonly measured using the accumulated amount of drugs delivered from the pharmacy to ward held stocks. The reliability of this method, particularly the impact of the length of the registration periods, has not been evaluated and such evaluation was aim of the study.

**Methods:**

During 26 weeks, we performed a weekly ward stock count of use of broad-spectrum antibiotics **- **that is second- and third-generation cephalosporins, carbapenems, and quinolones **- **in five hospital wards and compared the data with corresponding pharmacy sales figures during the same period. Defined daily doses (DDDs) for antibiotics were used as measurement units (WHO ATC/DDD classification). Consumption figures obtained with the two methods for different registration intervals were compared by use of intraclass correlation analysis and Bland-Altman statistics.

**Results:**

Broad-spectrum antibiotics accounted for a quarter to one-fifth of all systemic antibiotics (ATC group J01) used in the hospital and varied between wards, from 12.8 DDDs per 100 bed days in a urological ward to 24.5 DDDs in a pulmonary diseases ward. For the entire study period of 26 weeks, the pharmacy and ward defined daily doses figures for all broad-spectrum antibiotics differed only by 0.2%; however, for single wards deviations varied from -4.3% to 6.9%. The intraclass correlation coefficient, pharmacy versus ward data, increased from 0.78 to 0.94 for parenteral broad-spectrum antibiotics with increasing registration periods (1-4 weeks), whereas the corresponding figures for oral broad-spectrum antibiotics (ciprofloxacin) were from 0.46 to 0.74. For all broad-spectrum antibiotics and for parenteral antibiotics, limits of agreement between the two methods showed, according to Bland-Altman statistics, a deviation of ± 5% or less from average mean DDDs at 3- and 4-weeks registration intervals. Corresponding deviation for oral antibiotics was ± 21% at a 4-weeks interval.

**Conclusions:**

There is a need for caution in interpreting pharmacy sales data aggregated over short registration intervals, especially so for oral formulations. Even a one-month registration period may be too short.

## Background

Antibiotic use in hospitals accounts for 10% or less of total antibiotic consumption in most countries, but is characterized by the use of large quantities of broad-spectrum antibiotics (BSAs). Furthermore, hospital departments are densely populated with patients who are at particular risk of acquiring infections of resistant microorganisms [1,2]. Thus, active surveillance of in-hospital antibiotic use to prevent inappropriate prescribing is a fundamental measure in the struggle against the development of antimicrobial resistance [3,4].

The most common way of measuring antibiotic use in hospitals is to apply sales data extracted from hospital pharmacy computer systems. However, until now in most European countries pharmacies have not dispensed antibiotics directly to the patients. A ward-held stock of antibiotics has been the routine. In a recent survey of hospital pharmacy practice in Europe it was found that 70% of hospitals hold antibiotic stocks at the wards [5]. When wards purchase drugs from the pharmacy, there will necessarily be a time lag from ordering until consumption. Differences between sales figures and actual drug use might be expected because of variation of stock size over time, discarding of expired drugs, and exchanges of drugs between wards.

Moreover, the duration of the registration intervals may affect the results. In general, short registration intervals have been advocated, especially when the purpose has been to evaluate the effects of specific drug interventions [6-8]. However, the appropriateness of using short registration intervals has not been assessed. How the duration of the registration interval impacts the recording of antibiotic use has not been scrutinized.

Aim of the study was to explore whether the number of defined daily doses (DDDs) reported from the pharmacy, that is to say the sales data to the wards, reflects actual patient consumption of BSAs. In particular, we wanted to evaluate the importance of the length of the registration intervals for the reliability of the pharmacy data.

## Methods

### Study population

Oslo University Hospital Aker is a 350-bed tertiary hospital with adult surgical and medical specialities including regional functions for urology, vascular surgery, and endocrinology. In 2007, the number of somatic hospital beds was 356 and 20 060 patients were admitted for 116 251 days of in-patient treatment. Data on occupied bed-days were obtained from the hospital administration, where days of admission and discharge were counted together as one day.

Five hospital wards were included in the study: a pulmonary diseases ward, a combined gastrointestinal and infectious diseases ward, a combined endocrinology and haematology ward, and two urological wards. These wards accounted for 28% of all patient treatment in terms of occupied bed days. The specialties were selected because of a historically relatively high per-ward total use of BSAs, which in our hospital are ciprofloxacin (parenteral and oral) and parenteral formulations of cefuroxime, cefotaxime, ceftazidime, ceftriaxone, meropenem, and imipenem/cilastatin. Ciprofloxacin is the only oral BSA registered in Norway. Piperacillin+tazobactam is the only registered penicillin with an enzyme inhibitor, but this agent was not used in our hospital during the study period.

In Norway, a full assortment of antibiotics is normally stock-piled in the wards and administered by the ward nurses. Antibiotics are not ordered from the pharmacy on a per-patient basis. The ward nurses routinely prepare parenteral antibiotics, retrieved from the ward stock, just prior to administration. On rare occasions, when a drug is out of stock, it will be available from another ward or, in the daytime, from the hospital pharmacy. An emergency pharmacy service exists for essential and rarely used drugs; however, this does not apply for antibacterial agents.

### Ward stock accounting

During 26 weeks from October 2006 to April 2007, a pharmacist performed weekly counts of BSA stock in the five wards. The number of vials, infusion bags, and tablets were registered for each ward once a week, before daily orders of antibiotics were placed to the pharmacy, but after morning doses of antibiotics were administered to the patients. The milligram amounts for each antibiotic were converted to defined daily doses units in accordance with the latest WHO ATC/DDD version [9].

The weekly amount of antibiotics consumed by patients (henceforth designated as "ward BSA") was calculated as the difference between the previous and the current week count, and also taking into account input and output to ward stock. Factors increasing the ward stock size were delivery from the pharmacy and loan from other wards. Factors reducing the ward stock size were loan to other wards, BSAs sent with patients for use after discharge, and discarding of old drugs. Discarded drugs were registered electronically in the pharmacy sales system while the other stock reducing factors were registered manually by ward nurses.

Because of the lack of a ward stock count during holidays in study weeks 10 - 12 and 25 - 26, a weekly average was used for these weeks. Also, two weeks had to be omitted from a total of 26 weeks to establish complete 3- and 4-weeks registration intervals. We chose to omit the weeks 12 and 26, weeks with incomplete data acquisition, thus probably reducing the risk of error.

### Pharmacy sales data

For each ward, weekly sales figures for BSA DDDs for the same 26-week period were extracted from the hospital's pharmacy computer system (FarmaPro version 4.1.0a, NAF-data Corp. 2007, Oslo, Norway). In addition to economic data, the system registers the number of DDDs for each antibiotic order placed by the separate wards, as well as total amounts of antibiotics returned by the same wards to the pharmacy provided that shelf-life and condition allow for further use. The resulting sales figures for different time intervals, designated as "pharmacy BSA", were compared with "ward BSA". We chose to assign a weekly average for the study weeks 10 - 12 and 25 - 26 also for the pharmacy sales data (see Discussion). The shortest registration interval was one week, and accumulated periods of two, three, and four weeks were also investigated.

### Statistical analysis

The numbers of DDDs for each BSA and for each ward obtained by the two methods were entered into a Microsoft Excel (version 2007) spreadsheet. All entries and subsequent calculations were double-checked by two of the investigators (JBH and RM).

All statistical analyses were performed using Stata software version 11 (StataCorpLP, College Station, TX USA). The reliability or overall agreement of the pharmacy BSA with ward BSA was assessed by intraclass correlation coefficients (ICC) using a mixed model ANOVA. The theoretical formula for is ICC=*σ*_s_^2^+σ_e_^2^, where *σ*_s_^2 ^is the between-subject variance and *σ*_e_^2 ^is the within-subject variance. The ICC will be high if the measures are in agreement (i.e., the slopes of the regression lines are near 1), and the variation between ward data is large relative to the variation between pharmacy data measurements. A calculated ICC = 1 reflects perfect reliability of the method as evaluated against the assumed "gold standard". An ICC of 0.7 is commonly used as a threshold of sufficient reliability [10].

Using the method described by Bland and Altman [11,12], the differences between pairs of DDD measurements for pharmacy BSAs and for ward BSAs were plotted against their averages. Twelve plots (all antibiotics, parenteral antibiotics, and oral antibiotics assessed for one to four weeks registration intervals) were inspected for aberrant trends over the measurement range. Levels of agreement for different combinations were then determined and related to the average of mean DDDs for the two measurements.

## Results

### Use of broad-spectrum antimicrobial agents

BSA use during the 26 study weeks ranged from 12.8 DDDs/100 bed days in one urological ward to 24.5 DDDs/100 bed days in the pulmonary diseases ward. BSA amounts ranged from 615.3 DDDs at one urological ward to 1144.8 DDDs at the pulmonary diseases ward (Table 1), which represents 19.4% - 26.3% of the total consumption of all systemic anti-bacterial agents (ATC group J01). In the urology and endocrinology/haematology wards, ciprofloxacin was the predominant BSA used. In the pulmonary diseases ward, cephalosporins accounted for two-thirds of total BSA use, whereas the use of the various antibiotics was more evenly distributed in the gastroenterology/infectious diseases ward.

**Table 1:** Use of broad-spectrum antibiotics (BSAs) ^1 ^during 26 weeks, pharmacy sales data versus ward stock data.

**Wards **BSAs	PharmacyBSA DDDs ^2^	WardBSA DDDs	Diff. (%)
**Pulmonary diseases**	**1 144.8**	**1 194.2**	-**4.3**
2^nd ^generation cephalosporins	445.0	432.5	-2.8
3^rd ^generation cephalosporins	328.8	360.3	-9.6
Carbapenems	180.0	171.0	5.0
Ciprofloxacin parenteral	16.0	26.4	-65.0
Ciprofloxacin oral	175.0	204.0	-16.6
			
**Gastrointestinal/infectious diseases**	**877.3**	**898.1**	-**2.4**
2^nd ^generation cephalosporins	205.0	231.0	-12.7
3^rd ^generation cephalosporins	161.3	152.5	5.5
Carbapenems	103.0	96.3	6.5
Ciprofloxacin parenteral	128.0	130.0	-1.6
Ciprofloxacin oral	280.0	288.3	-3.0
			
**Endocrinology/haematology**	**668.3**	**676.1**	-**1.2**
2^nd ^generation cephalosporins	125.0	128.0	-2.4
3^rd ^generation cephalosporins	101.3	111.3	-9.9
Carbapenems	80.0	76.0	5.0
Ciprofloxacin parenteral	52.0	46.8	10.0
Ciprofloxacin oral	310.0	314.0	-1.3
			
**Urology 1**	**615.3**	**572.8**	**6.9**
2^nd ^generation cephalosporins	70.0	62.5	10.5
3^rd ^generation cephalosporins	46.3	32.8	29.2
Carbapenems	0	0	-
Ciprofloxacin parenteral	124.0	131.2	-5.8
Ciprofloxacin oral	375.0	346.3	7.7
			
**Urology 2**	**671.5**	**628.7**	**6.4**
2^nd ^generation cephalosporins	72.5	63.0	13.1
3^rd ^generation cephalosporins	30.0	19.0	36.7
Carbapenems	0	0	-
Ciprofloxacin parenteral	144.0	144.4	-0.3
Ciprofloxacin oral	425.0	402.3	5.3

**All specialties**	**3 977.2**	**3 969.7**	**0.2**

^1^BSAs: ciprofloxacin (oral and parenteral), cefuroxime, cefotaxime, ceftazidime, ceftriaxone, meropenem and imipenem/cilastatin

^2^DDD*: *defined daily doses

### Pharmacy versus ward DDD registrations

During the 26 study weeks there were 1040 dual registrations (pharmacy versus ward data) of the eight BSAs at the five wards. In 550 of these registrations, no use was registered by either of the two methods and this was primarily caused by a very low use of carbapenems (e.g. no use in the urological wards). The number of DDDs that were discarded (10.5), sent with patients on discharge (18.0) or loaned to other wards (14.5) represented 1.1% of the total consumption. Only nine DDDs were borrowed from other wards.

**Figure 1:**
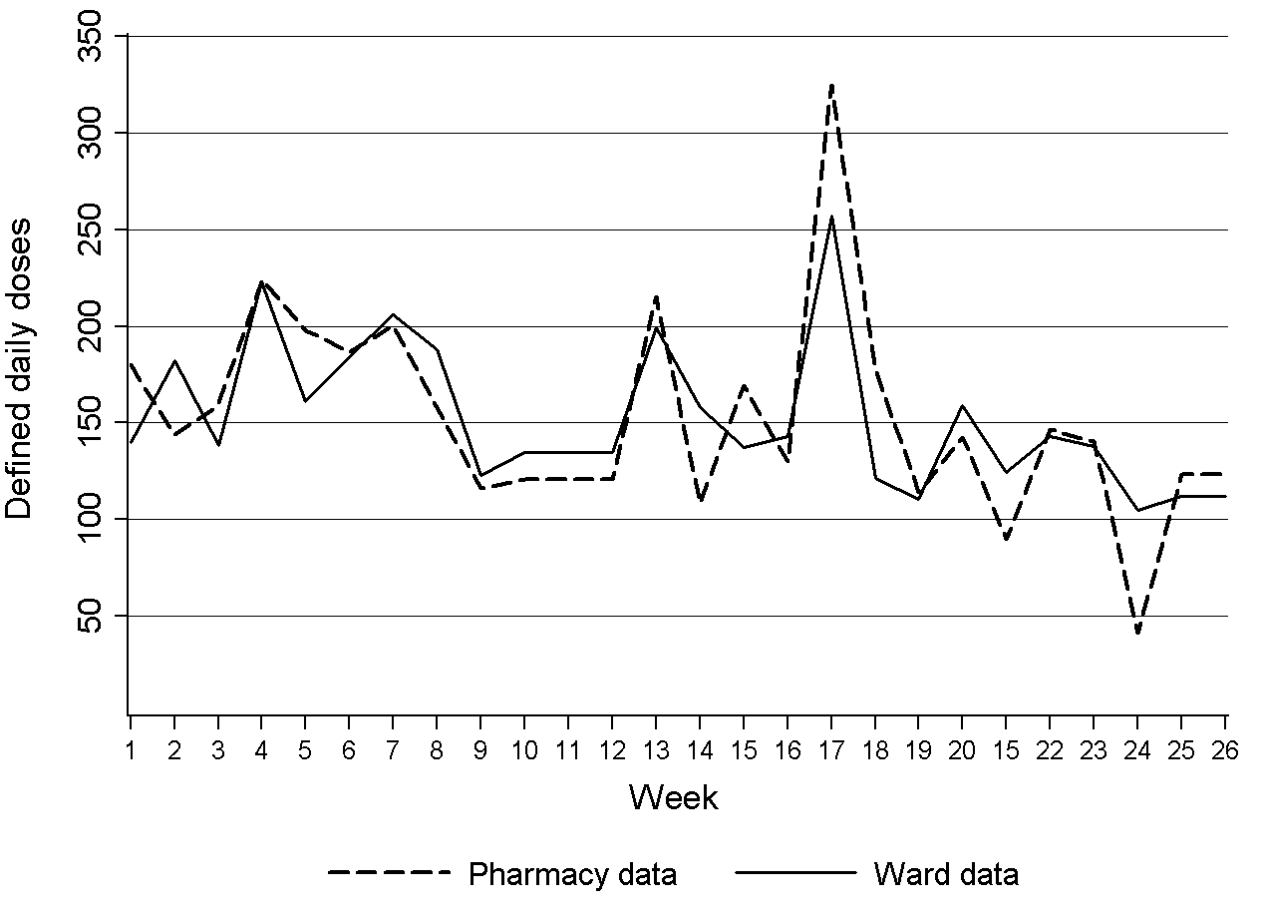
**Consumption of broad-spectrum antibiotics in defined daily doses (DDDs) during 26 weeks; pharmacy sales data versus ward stock measurements (all wards)**.

Total BSA use over 26 weeks measured as ward BSA was 3 970 DDDs with a weekly range of 104.7 - 257.2 DDDs. The corresponding total number for pharmacy sales data were almost identical at 3 977 DDDs, with a weekly range of 41.5 - 324.5 DDDs (Figure 1). The largest discrepancy between total ward and pharmacy BSAs was noted in observation weeks 17 - 18 and was related to oral ciprofloxacin. Half of the total BSA use in this study was found to be ciprofloxacin and 77% of this was oral formulation. In both urological wards, the total pharmacy BSA was markedly higher than ward BSA (6.4% - 6.9%). This is in contrast to the three medical wards where the total pharmacy BSA was somewhat lower than ward BSA (1.2% - 4.3%).

**Figure 2:**
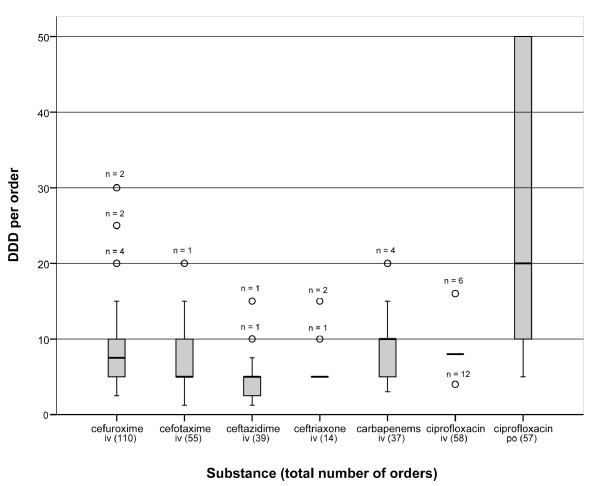
**Antibiotic orders ^1 ^from wards to the pharmacy for different broad-spectrum antibiotics ^2 ^during 26 weeks (all wards)**. **^1 ^**Median number of defined daily doses per order, interquartile ranges and outliers (circles). **^2 ^**Carbapenems include meropenem and imipenem/cilastatin.

The pharmacy sale to the wards per individual order of antibiotics varied little for the various parenteral formulations (median 5 - 10 DDDs) as distinct from oral formulations (ciprofloxacin tablets) for which both larger bulks and a wider range of order size (5 - 50 DDDs, median 20) were registered (Figure 2). The total number of orders varied considerably from 14 (ceftriaxone) to 110 (cefuroxime).

### Reliability analysis

The reliability of pharmacy data as compared to ward stock accounting increased with longer surveillance intervals (Table 2). For total BSAs, all wards included, an intraclass correlation coefficient (ICC) of 0.65 (CI 0.61 - 0.69) was found for one-week intervals whereas for 4-week registration intervals the ICC was 0.90 (CI 0.88 - 0.93). The reliability of pharmacy data was markedly higher for parenteral than for oral BSAs, the latter achieving an ICC of 0.74 (CI 0.58 - 0.90) only at four-week registration, whereas parenteral BSAs had already reached this level at the one-week registration interval.

**Table 2:** Reliability of pharmacy sales compared with ward stock data of broad-spectrum antibiotics (BSAs) ^1 ^for different registration intervals

Interval	**No. reg**.	ICC ^2 ^all BSA (CI ^3^)	ICC parenteral BSA (CI)	ICC oral BSA (CI)
1 week	1 040	0.65 (0.61-0.69)	0.78 (0.76 - 0.81)	0.46 (0.32 - 0.59)
2 weeks	520	0.77 (0.74 - 0.81)	0.87 (0.85 - 0.89)	0.53 (0.35-0.70)
3 weeks	360	0.82 (0.79-0.86)	0.93 (0.92 - 0.95)	0.54 (0.32-0.76)
4 weeks	280	0.90 (0.88-0.93)	0.94 (0.92 - 0.95)	0.74 (0.58 - 0.90)

^1 ^Broad-spectrum antibiotics: ciprofloxacin (parenteral and oral), cefuroxime, cefotaxime, ceftazidime, ceftriaxone, meropenem, and imipenem/cilastatin

^2 ^ICC: intraclass correlation coefficient

^3 ^CI: 95% confidence interval

**Figure 3:**
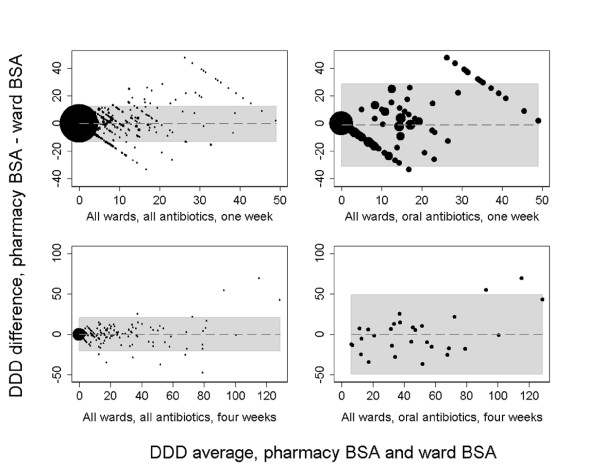
**Accumulated data for antibiotic use at all wards: one-month and four months registration intervals of all BSAs ^1 ^and oral BSAs (Bland-Altman plots ^2 ^with limits of agreement in gray shade)**. ^1 ^Broad-spectrum antibiotics. ^2 ^See reference 13.

For all of the 12 combinations of antibiotics (all, parenteral, oral) and periods of registrations (one to four weeks), Bland-Altman statistics revealed mean differences between -0.136 to 0.158 DDDs. The limits of agreement for each of the above combinations were converted to corresponding DDD ranges (Table 3). For all BSAs and parenteral BSAs, limits of agreement of < ± 5% were found for 3- and 4-weeks registration intervals while oral antibiotics (ciprofloxacin) deviated ± 21% from the average mean DDD use even at the 4-week registration interval. Bland-Altman plots (Figure 3) showed diverging differences with increasing averages of DDDs for oral BSAs and for the shorter registration intervals in general; a trend which was far less pronounced for parenteral BSAs and all BSAs at the 4-weeks intervals.

**Table 3:** Mean average DDD use ^1 ^of all broad-spectrum antibiotics and corresponding limits of agreement ^2 ^(DDD use range) for different registration intervals (all wards combined).

Interval	Defined daily doses (DDDs)		
	
Antibiotics	Mean use	Use range (- %; + %)	Mean difference
1 week			
All	152.8	144.1 - 167.5 (9.6; 9.6)	0.007
Parenteral	92.8	85.0 - 100.6 (8.4; 8.4)	-0.003
Oral	60.0	23.9 - 96.2 (60.2; 60.4)	0.079
2 weeks			
All	305.6	287.1 - 324.1 (6.1; 6.1)	0.014
Parenteral	185.6	175.5 - 195.7 (5.5; 5.4)	-0.006
Oral	120.0	74.9 - 165.4 (37.6; 37.8)	0.158
3 weeks ^3 ^			
All	466.0	442.8 - 489.2 (5.0; 5.0)	0.031
Parenteral	288.7	274.5 - 294.9 (3.5; 3.6)	0.050
Oral	181.3	120.7 - 241.7 (33.4; 33.3)	-0.102
4 weeks ^3 ^			
All	621.3	600.6 - 642.1 (3.3; 3.3)	0.042
Parenteral	379.6	367.6 - 391.8 (3.2; 3.2)	0.067
Oral	241.7	191.6 - 291.5 (20.7; 20.6)	-0.136

^1 ^Sum of averages of paired results (pharmacy sales DDDs and ward stock DDDs)

^2 ^Limits of agreement according to Bland-Altman statistics are expressed as the range of DDDs and percent DDD deviation below and over the mean average use

^3 ^Weeks 12 and 26 were discarded (see Methods)

## Discussion

Accurate information regarding antibiotic consumption is a prerequisite for evaluating antibiotic use and implementing measures to avoid excessive prescribing and increased bacterial resistance. To achieve efficient monitoring, short registration periods have been advocated, preferably as short as one month [13]. In this study, we found that surveillance by use of pharmacy sales data was sufficiently reliable for the total registration period of six months. A small mean difference between measurements for all registration intervals, as demonstrated by Bland-Altman statistics, implies that our comparison of pharmacy with ward registrations was not burdened with systematic bias. However, when data for the shorter periods, such as one to four weeks, were investigated, ICCs indicated that pharmacy sales data were not sufficiently reliable, particularly so for the one- to three-week registrations. It will be a clinical decision to define acceptable limits of agreement. Nonetheless, the wide DDD range for oral BSAs (± 21%) even at a four weeks registration interval seems unacceptable by any standards.

The amount of BSAs exchanged between wards, discarded due to exceeded durability or given to patients at discharge was less than 2%, and it was the stock size fluctuations in the wards that accounted for the main discrepancy between pharmacy and ward data. The greatest variations were observed for ciprofloxacin tablets, which largely explain the less reliable pharmacy figures for oral compared to parenteral formulations. This in turn is related to the fact that tablets usually are less voluminous and also cheaper per DDD and may be stored for longer periods than injectable preparations.

Few other studies have evaluated the common method of measuring hospital use of antibiotics by recording pharmacy dispensing or sales data and, to our knowledge, none has previously evaluated the impact of the length of the registration interval. One short report found a poor correlation between a pharmacy dispensing system and an intensive care unit (ICU) based electronic chart as source of data. The investigators speculated that transfer of antibiotics between wards, wastage, and data-entry errors may have been the reasons for the discrepancy [14]. Another study compared pharmacy sales data, based on pharmacy dispensing, with data from nursing records and found that up to 20% of parenteral doses of antibiotics dispensed at the pharmacy were not administered to patients [15]. Such a marked deviation is probably due to delayed transmission of drug information between the ward and the pharmacy, a situation that arises when parenteral antibiotics are prepared at the pharmacy and not near the patient. Neither of the studies evaluated the length of the registration period.

A limitation of our study is the relatively low BSA consumption in our institution, such that the number of registered DDDs was moderate. In hospitals with more extensive BSA consumption and a larger assortment of antibiotic substances, the findings may differ. Also of note, for two periods weekly ward data were missing because of holidays, and for the weeks in question, weekly averages were the basis for the analyses. However, since the corresponding pharmacy sales data were averaged accordingly, this deviation tend to introduce bias towards higher levels of agreement (alpha error), particularly so for the short registration intervals.

We propose that our method of weekly ward stock DDD accounting is an accurate method for indicating actual antibiotic use by patients. Only a much more labour-intensive patient chart review would be more accurate. Although also our method is demanding and therefore not practical for routine surveillance, we regard it as well suited for scientific purposes and for quality assessments of pharmacy sales figures. In some hospitals, electronic patient charts have been introduced and this allows for another method, probably with a high level of accuracy, for measuring drug consumption [16]. However, for the immediate future in most European hospitals, the pharmacy will remain the principal data source for surveillance of use of antibiotics.

## Conclusions

Pharmacy sales data for total BSA use were representative for the actual drug consumption when longer registration periods were used. For the data to be sufficiently reliable, a four-week registration period is required for parenteral formulations, whereas for oral medications one month is not sufficient. For analyses of BSA subclasses and separate hospital units, even these intervals are probably too short, at least in hospitals with a low-to-moderate consumption profile.

## Competing interests

The authors declare that they have no competing interests.

## Authors' contributions

JBH designed the study, analyzed and interpreted the data and drafted the manuscript. RM contributed to study design, acquired the ward stock data and helped in interpretation and writing of the manuscript. ÅR advised on design and interpretation of the study, and contributed significantly with critical revisions of the manuscript.

All authors have read and approved of the final manuscript.

## Pre-publication history

The pre-publication history for this paper can be accessed here:

http://www.biomedcentral.com/1471-2288/11/166/prepub
